# Low Serum B12 Concentrations Are Associated with Low B12 Dietary Intake But Not with *Helicobacter pylori* Infection or Abnormal Gastric Function in Rural Mexican Women

**DOI:** 10.3390/nu11122922

**Published:** 2019-12-02

**Authors:** Miriam A. Anaya-Loyola, Alex Brito, Haydé Vergara-Castañeda, Carina Sosa, Jorge L. Rosado, Lindsay H. Allen

**Affiliations:** 1 Escuela de Nutrición, Facultad de Ciencias Naturales, Universidad Autónoma de Querétaro, Querétaro, México, Av de las Ciencias S/N, Santiago de Querétaro, QT 76230, Mexico; aracely.anaya@uaq.mx (M.A.A.-L.); casoal08@yahoo.com.mx (C.S.); jlrosado@prodigy.net.mx (J.L.R.); 2 Laboratory of Pharmacokinetics and Metabolomic Analysis. Institute of Translational Medicine and Biotechnology. I.M. Sechenov First Moscow State Medical University, 2-4 Bolshaya Pirogovskaya St., 119991 Moscow, Russia; abrito@labworks.ru; 3 Luxembourg Institute of Health, Department of Population Health, NutriHealth Group, 1 A-B, rue Thomas Edison, L-1445 Strassen, Luxembourg; 4 Facultad de Medicina, Universidad Autónoma de Querétaro, Querétaro, México, Clavel 200, Prados de La Capilla, Santiago de Querétaro, QT 76176, Mexico; hayde.vergara@uaq.mx; 5 USDA, ARS Western Human Nutrition Research Center, 430 W. Health Sciences Drive, University of California, Davis, CA 95616, USA

**Keywords:** vitamin B12, deficiency, diet, *Helicobacter pylori*, gastric function, Mexico

## Abstract

Background: Gastric function, *Helicobacter pylori* infection, and vitamin B12 (B12) dietary intake were assessed as predictors of serum B12. Methods: *H. pylori* antibodies, gastric function, B12 dietary intake, and biochemical/hematological parameters were measured in 191 adult women from two rural communities in Querétaro, Mexico. Results: The overall mean serum B12 concentration was 211 ± 117 pmol/L. The prevalences of low (≤ 148 pmol/L), marginal (148 to 221 pmol/L), and adequate (> 221 pmol/L) serum B12 were 28.4%, 31.1%, and 40.5%, respectively. Seventy-one percent of women tested positive for *H. pylori* antibodies. The prevalence of gastric function categories did not differ by serum B12 categories. The odds ratio for having low serum B12 was 2.7 (*p* = 0.01) for women with an intake below the estimated average requirement, 3.6 (*p* = 0.01) for those in the lowest tertile of total B12 intake, and 3.0 (*p* = 0.02) for those in the lowest tertile of B12 intake from animal source foods. Age and B12 intake were predictors of serum B12 concentrations [serum B12 (pmol/L) = 90.060 + 5.208 (B12 intake, µg/day) + 2.989 (age, years). Conclusions: Low serum B12 concentrations were associated with low B12 dietary intake but not with *H. pylori* infection or abnormal gastric function in rural Mexican women.

## 1. Introduction

A high prevalence of vitamin B12 (B12) deficiency has been identified in previous studies in rural Mexico [1,2]. There is little doubt that B12 deficiency, once believed to be associated with cohorts of strict vegetarians and patients with pernicious anemia, is commonly encountered in many Latin American countries [3]. The main causes of B12 deficiency may be a low intake of animal source foods (ASFs) and/or malabsorption due to atrophic gastritis, e.g., resulting from *Helicobacter pylori* infection [4]. Most of the studies on causes of B12 deficiency where gastric function was assessed were conducted in the elderly [5,6,7]. The higher prevalence of B12 deficiency in the elderly populations is believed to be the result of malabsorption of food-bound B12 related to gastric atrophy associated with aging [4]. In the case of healthy adults with B12 deficiency, it is expected that deficiency originates from dietary intake inadequacy [8]. However, in populations such as those in rural Mexico, it is well known that *H. pylori* infection and bacterial overgrowth are common [9]. Long-term infection with *H. pylori* can cause gastric atrophy, leading to poor secretion of gastric acid (intrinsic factor) with accompanying malabsorption of B12 from food [9]. Thus, the goal of the present study was to assess gastric function with serum gastrin and pepsinogen I (PG-I) as well as antibodies to *H. pylori* as effectors of serum B12 concentrations. Associations between serum B12 levels, dietary intake of the vitamin, and the age of the participants were also examined.

## 2. Participants and Methods

### 2.1. Recruitment

Adult women from two rural communities (La Fuente and Los Cerritos) located in the state of Querétaro (approximately 200 km north of Mexico City and 45 km from the city of Querétaro) were recruited after advertising the study throughout the health system and local media, with prior approval by local authorities. The study purpose and procedures were described by the field supervisor at the health or community center, and those who volunteered to participate signed an informed consent form. Eligibility criteria included age > 17 years and the absence of visible health problems. Reported chronic disease, pregnancy or lactation, and current or recent consumption of micronutrient supplements were exclusion criteria. This research was executed after being approved by the Human Research Committee at the Universidad Autónoma de Querétaro (UAQ) in Mexico (Protocol ID#FNN−2004−03) and by the Institutional Review Board at the University of California, Davis (UC Davis). The study was performed in conformity with the ethical principles for medical research involving humans stated in the Declaration of Helsinki.

### 2.2. Data Collection

During the first visit to the health center or community clinic, volunteers were interviewed and data obtained on their socio-economic status (SES), medical history, blood pressure, anthropometry, and diet. SES information was collected by trained field workers and included: demographic information (i.e., number of household members, household crowding calculated as household members/number of rooms); education (years of schooling); occupation of the participant; and income of the household.

### 2.3. Anthropometric Data

Body weight was measured to the nearest 0.1 kg (SECA model 843 digital scale, Hamburg, Germany), in duplicate. If the difference between duplicates was ≥ 0.3 kg, a third measurement was taken. Height was measured in duplicate to the nearest 0.1 cm with a stadiometer (SECA model 208, Hamburg, Germany). A third measure was taken if the duplicates differed by ≥ 0.5 cm. Body mass index (BMI) was calculated using the formula: weight in kg/height in m^2^, and classified as underweight or normal (≤ 25 kg/m^2^), overweight (25 to 30 kg/m^2^), or obese (≥ 30 kg/m^2^) [10].

### 2.4. Sample Collection and Laboratory Assessment

On a second visit to the health center or clinic, a fasting venous blood sample (10 mL) was collected in silicone-coated BD vacutainer tubes, with and without added EDTA. Immediately after collection, the blood was stored in a cooler on ice and transported to the UAQ for processing and storage. The tubes designated for serum were left at room temperature for 30 min to allow clotting after complete blood count (CBC), and the remaining blood samples were spun at 1500 g for 20 min in a refrigerated centrifuge (Precision 300R, Thermo Electron Corporation, Chateau Gontier, France). Serum and plasma aliquots were separated and stored in microvials at −70 °C at the UAQ. Samples were later transported on dry ice to the U.S. Department of Agriculture (USDA), ARS Western Human Nutrition Research Center (WHNRC) in Davis, CA, USA, and stored at −70 °C. Hematological analysis and *H. pylori* infection detection were carried out at the UAQ. Biochemical and serum gastric function analyses were carried out at the WHNRC.

### 2.5. Biochemical and Hematological Analyses

Serum B12 and folate were determined in duplicate by radioassay (Simultrac radioassay kit vitamin B12 (^57^ Co/Folate ^125^I), ICN Pharmaceuticals, Orangeburg, NY USA), using control serum samples in each run. Serum B12 concentrations were defined as low (≤ 148 pmol/L), marginal (148−221 pmol/L) or adequate (> 221 pmol/L) [8]. The serum folate cut-off for low values was set at < 10 nmol/L [8]. Serum ferritin < 12 µg/L was classified as iron deficiency. A 50-µL sample of whole blood was used for a CBC analysis by a hematological cell counter (CellDyn 1400; Abbott, Chicago, IL, USA). Cut-offs for CBC values were: hemoglobin (Hb) < 127 g/L and hematocrit (Hct) < 38%−47% (both corrected for the 1846-m altitude of the two communities) [11]; mean cell volume (MCV) ≤ 80 fL for microcytosis and > 96 fL for macrocytosis; mean corpuscular hemoglobin (MCH) < 27 pg (hypochromia) and > 33 pg (hyperchromia); mean corpuscular hemoglobin concentration (MCHC) < 30 g/dL (hypochromia) and > 36 g/dL (hyperchromia); normal red blood cell (RBC) count 4.2−5.4 × 10^6^ cells/µL; normal white blood cell (WBC) count < 4.5−11.0 × 10^3^ cells/µL; normal platelet count 150−450 × 10^3^ cells/µL; and normal lymphocyte count, 1.5−4.0 cells/µL [12].

### 2.6. Gastric Function and *H. pylori* Infection

Serum gastrin was assessed by the Gastrin-RIA double antibody test (DPC, Inc., Orangeburg, NY). Gastrin concentrations > 90 pg/mL were considered indicative of gastric mucosal inflammation or achlorhydria [13]. Pepsinogen I (PG-I) was determined in a 1:10 dilution of serum by ELISA (Pepsinogen I kit, Bio-Hit, PLC, Helsinki, Finland). PG-I concentrations < 20 µg/L were considered indicative of gastric atrophy function and those > 80 µg/L were defined as elevated [13]. Serum Immunoglobulin G (IgG) antibodies to *H. pylori* were quantified by EIA (ImmunoComb II Helicobacter pyloriG, Anti H. Pylori EIA, Orgenics, Ltd., Yavne, Israel). A seropositive response to *H. pylori* antibodies was detected based on a colored line that becomes visible in the test strip, according to the manufacturer’s recommendations. Gastric function categories were defined based on the algorithm used by the Gastrosoft software (Gastrosoft, Medtronic Synectics, Irving, TX, USA) as: “normal gastric function” if there was a negative response to *H. pylori* antibodies plus normal gastrin and normal PG-I; “non-atrophic *H. Pylori* infection” if there was a positive *H. pylori* antibody response plus abnormal gastrin but normal PG-I; “atrophic gastritis of the antrum” if there was abnormal gastrin; “atrophic gastritis of the corpus” if there was abnormal PG-I; and “atrophic pangastritis” (atrophic gastritis of the antrum and corpus) if both gastrin and PG-I were abnormal.

### 2.7. Dietary Intake Estimation

Usual dietary intake was estimated with a food frequency questionnaire that focused on intake of animal source foods (ASFs) and other potential sources of B12, including fortified cereals and supplements, in the last 30 days. The dietary databases developed by the U.S. Department of Agriculture (USDA, 2011) [14] were used. Dietary intake estimations were also complemented with Mexican and Central American food databases [15]. The cut points used to classify dietary intake adequacy were based on the Estimated Average Requirements (EAR = 2.0 µg/day) and the Recommended Dietary Allowance (RDA = 2.4 µg/day) for B12 calculated for adults by the Institute of Medicine [16].

### 2.8. Statistical Methods

The normality of the variables was tested using the Shapiro–Wilk test. The normally-distributed variables were expressed as mean (SD) and the categorical variables as frequencies (%). Skewed variables (i.e., serum B12, gastrin, ferritin, and PG-I) were log or square transformed. One-way ANOVA was used to compare means among the three serum B12 categories (low, marginal, and adequate) for general characteristics and biochemical, hematological, and serum gastric function parameters. Bonferroni’s method was used as a post hoc test for multiple comparisons. The Chi-squared test was used to compare differences in percentages among groups with the Fisher’s least significant difference procedure as a post hoc test for multiple comparisons. Pearson’s correlations were run between log serum B12 versus age, weight, height, BMI, serum folate, gastric function markers (gastrin and PG-I), ferritin, food intake (B12, servings and intake of energy and protein from ASF), hematological values (Hb, Hct, MCV, MCH, RBC, MCHC), and socioeconomic factors (number of household members, children, years of schooling). Logistic regressions were run defining the low and marginal serum B12 categories as dependent variables versus B12 dietary intake above and below the EAR, between tertiles of total B12 intake, and between tertiles of B12 intake from ASFs as independent variables. Forward stepwise regressions were performed to identify the variables predicting serum B12 concentrations. A *p*-value < 0.05 was considered statistically significant. Statistical analyses were conducted using STATVIEW (version 5.0.1, SAS Institute Inc., Cary, NC) and the R software (R Development Core Team, 2008).

## 3. Results

### 3.1. General Characteristics of the Women

The average age of the women (*n* = 191) was 36.2 ± 12.4 years (17−74 years). The mean BMI was 28.9 ± 6.2 kg/m^2^, with most overweight (25%) or obese (44%). Stratifying general characteristics by serum B12 categories showed that most of the other variables were not significantly different across the low, marginal, and adequate B12 groups with the exception of age, parity, and education (Table 1). Log serum B12 concentrations were directly correlated with age (r = 0.25, *p <* 0.001) and inversely correlated with years of education (r = −0.017, *p* = 0.01) (data not shown). Log serum B12 concentrations were not correlated with weight or BMI (data not shown). Women with adequate B12 status were about 5 years older than the deficient and marginal groups, but had fewer years of education.

### 3.2. Biochemical and Hematological Parameters

**Figure 1:**
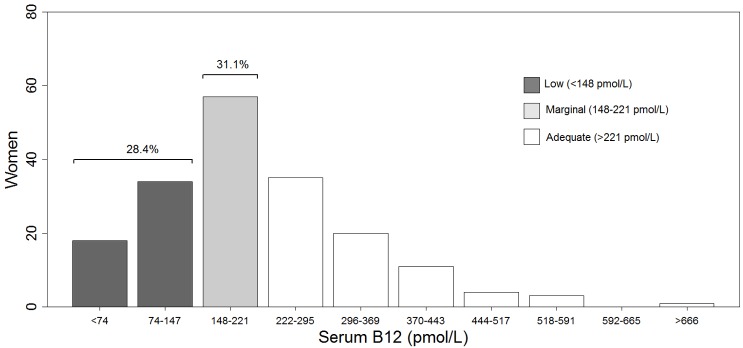
Prevalence of low, marginal, and adequate serum B12.

The overall mean serum B12 concentration was 211 ± 117 pmol/L. The prevalences of low (≤ 148 pmol/L), marginal (148 to 221 pmol/L), and adequate (>221 pmol/L) serum B12 values were 28.4%, 31.1%, and 40.5%, respectively (Figure 1). The mean serum folate concentration was 17.4 ± 6.7 nmol/L. Only 6.3% presented with low serum folate (< 10 nmol/L). There was a wide range of serum ferritin concentrations, but there were no significant differences in serum ferritin or in the prevalence of iron deficiency (serum ferritin < 12 µg/L) across serum B12 categories (Table 2). Serum B12 and ferritin concentrations were weakly correlated (r = 0.154, *p* < 0.05) (data not shown). Although the mean Hb concentration was 135.7 ± 17.2 g/L, 12% of the women were anemic (*n* = 23). Hb concentration was lower (*p* < 0.05) in the low B12 group versus the marginal and adequate groups, but there was no difference in the prevalence of anemia across groups. The rest of the CBC parameters with the exception of WBC count were not significantly different across serum B12 categories. Differences in the WBC count varied inconsistently with serum B12 categories. The overall prevalences of low, normal, and high Hct values were 11%, 74%, and 15%, respectively. Macrocytosis was present in 5% of the women, and microcytosis in 12%, of whom 80% also had a low Hct. RBC count was normal in 89% of the participants, while 8% presented a slightly high count. Few (7%) had a low WBC count or an abnormal number of platelets (5%), and none had an abnormal lymphocyte count (Table 2).

### 3.3. Gastric Function and *H. pylori* Infection

High serum gastrin concentrations (> 90 pg/mL) were detected in 24% of the women, and abnormal PG-I values in 23%, of which 4% were low (< 20 µg/L) and 19% were high (> 80 µg/L). Serum gastrin was lower (*p* < 0.05) in the low B12 group than in the marginal and adequate groups, while PG-I presented the highest concentration in the B12 marginal group. The majority (71%) of women were positive for *H. pylori* antibodies. The gastric function categories did not differ significantly across serum B12 categories. Normal gastric function was present in 19%, 27%, and 22%; and non-atrophic *H. pylori* infection in 46%, 49%, and 49% of women with low, marginal, and adequate B12, respectively. The prevalence of atrophic gastritis of the antrum and of the corpus was very low (≤ 2%) across B12 categories. Atrophic pangastritis was present in 33%, 24%, and 31% of women with low, marginal, and adequate serum B12, respectively (Table 3). Mean serum B12, gastrin and PG-I concentrations did not differ between the *H. pylori*-seropositive and -seronegative women (data not shown).

### 3.4. Dietary Intake Estimation

The mean total energy intake was 2854 ± 797 kcal/d, with maize tortillas (822 kcal/d) and beans (298 kcal/day) the main contributors. Soft drink (219 kcal/d), tomato (175 kcal/d), and vegetable oil (191 kcal/day) consumption were also important sources of energy. On average, 2.4 ± 1.1 servings of ASFs were estimated to be consumed daily. B12 intake averaged 3.8 ± 3.5 µg/day, of which approximately 95% came from ASFs (primarily beef and chicken liver, dry milk, and eggs) with the remaining ~5% obtained from fortified cereals and cocoa powder. When diets were compared across serum B12 categories, women in the low B12 category had a significantly lower consumption of ASF servings than the B12 adequate group, and significantly (~1.5 µg/day) less B12 intake than the marginal or adequate B12 groups (Table 4). Sixty-three percent of the women in the low B12 group had an estimated B12 intake below the RDA (2.4 µg/day) and 34% below the EAR (2.0 µg/day). Serum B12 concentrations were higher when estimated intakes of the vitamin were above the RDA or above the EAR. The proportion with low, marginal and adequate B12 status was different (*p* < 0.05) for those with a B12 intake above versus below the RDA (data not shown). The odds ratios for having low serum B12 concentrations were 2.7 (*p* = 0.01) for women with an intake below the EAR, 3.6 (*p* = 0.01) for those in the lowest tertile of total B12 intake, and 3.0 (*p* = 0.02) for those in the lowest tertile of B12 intake from ASFs (Table 5). Log-transformed serum B12 concentrations were directly correlated with total dietary B12 intake (r = 0.21, *p <* 0.005), servings of ASF (r = 0.18, *p <* 0.05), grams of ASF (r = 0.26, *p* ≤ 0.001), and µg B12 intake from ASF (r = 0.21, *p* < 0.005) (data not shown).

### 3.5. Predictors of Serum Vitamin B12

Forward stepwise regression to identify predictors of serum B12 concentration included age, serum gastrin, PG-I, serum folate and ferritin, and B12 intake. The final linear model found age and B12 intake to be the significant predictors of serum B12 concentrations as follows: serum B12 (pmol/L) = 90.060 + 5.208 (B12 intake, µg/day) + 2.989 (age, years). The age ranges were 18–59 years, 17–74 years, and 20–74 years in the low, marginal, and B12-adequate groups- while the ranges of B12 dietary intake for each group were 0.46−25.46 µg, 0.11−22.38 µg- and 0.59−37.65 µg, respectively.

## 4. Discussion

According to the 2006 and 2012 National Nutrition Surveys, Mexico has experienced a reduction in the prevalence of B12 deficiency that can be explained by the success of national policies to improve micronutrient status [17,18]. In this 2004 study almost 60% of the women had low or marginal serum B12 concentrations. This high prevalence of low B12 status expands previous data reported in Mexico around those years [1,2], including the first Mexico National Nutrition Survey (1999) where 46.6% adult women and 57.8% of adolescent girls had low or marginal B12 values [19]. In this study, we demonstrated that B12 intake was a stronger predictor of serum B12 concentrations than gastric function in a rural population of Mexican women.

*H. pylori* infection is frequent in low-income countries, and 71% of the women in this study were positive for *H. pylori* antibodies. In wealthier countries, at the time of execution of this study the fraction of infected individuals < 40 years was around 20% [20]. A positive immunological reaction is indicative of current or recent infection (approximately in the past 6 months). The initial stages of infection are characterized by gastric inflammation and greater acid secretion from the gastric mucosa. Over time, usually many years, infected individuals are at risk of developing gastric atrophy with low gastric acid secretion, and if severe, eventual lack of intrinsic factor, a glycoprotein necessary for the absorption of B12. *H. pylori* infection is thought to be the main cause of the gastric atrophy that affects about one-third of the elderly in the United States [21]. It is well-known that achlorhydria can impair the release of B12 from food proteins, and potentially cause malabsorption of B12 bound to its food matrix; however, the causal role of *H. pylori* infection in food-bound malabsorption is uncertain [20] and possibly complex. *H. pylori* infection is often present for many years without leading to symptoms of gastric damage, and different genotypes have shown different manifestations [22]. Nevertheless, 78% of B12-deficient elderly diagnosed with malabsorption of B12 from food had serum antibodies against *H. pylori,* compared to 42% of the elderly with normal B12 absorption [20]. Also, in 145 patients with *H. pylori* infection diagnosed by biopsy of the gastric mucosa, the eradication therapy resulted in increases in serum B12 [23]. Since the density of gastric infestation with *H. pylori* could not be determined in the present study, it is possible that severe infection did contribute to B12 depletion in some individuals. Another difficulty when attempting to relate the presence of *H. pylori* infection to B12 status is that some of these women may have had life-long infection, while in others infection may have occurred relatively recently and therefore did not result in gastric atrophy.

Considering the difficulties of linking *H. pylori* infection to B12 status, a better approach may be to assess direct gastric damage, since only when this progresses to gastric atrophy is it likely that B12 absorption from food is impaired. However, measuring direct gastric damage is difficult. Elevated levels of serum gastrin have been associated with food-bound B12 malabsorption [24]. In the present study, 24% of the women had high serum gastrin, but surprisingly gastrin concentrations were lower in the low serum B12 group. This is the opposite relationship to that which we reported in a representative sample of 183 older (> 60-year) Latinos in Sacramento, California; 48% of those with serum B12 < 148 pmol/L had elevated serum gastrin compared to only 23% with marginal depletion (148−221 pmol/L) and to 21% of those with adequate status (≥ 221 pmol/L) [7].

It appears that these younger Mexican women did not develop the more severe gastric atrophy that occurs in the elderly, and for those who had gastric function impairment it was only at the level of gastric inflammation (gastritis), the earlier stage of gastric reaction to *H. pylori* infection [25]. Two-thirds of the *H. pylori*-positive women had non-atrophic gastritis, based on elevated serum gastrin, but normal PG-I. Gastritis of the antrum and corpus (pangastritis) affected approximately 30% of the total and around 20% of *H. pylori*-positive women. In gastritis, parietal cell function is compromised, reducing secretion of PG-I and PG-II, acid release, and the production of intrinsic factor [26,27]. However, low serum PG-I concentrations were found in only 4% of these women, again supporting that they had gastric inflammation which had not yet developed to gastric atrophy. We did not measure PG-II limiting our ability to do a more accurate classification of serum gastric function impairment.

Kaptan et al. did report that eradication of *H. pylori* infection improved B12 status in adults with low serum B12 (< 148 pmol/L), suggesting that *H. pylori* infection is a causative agent in the development of B12 deficiency in adults [28]. The discrepancy of these findings with our results might be explained by the better B12 status of our population, and also by weakness in our definition of gastric function. Kaptan et al. used upper gastrointestinal endoscopy to assess the severity of atrophic gastritis and histological examination for *H. pylori* infection [28]. It would have been ideal to have gastrointestinal endoscopy and/or gastric biopsies as confirmatory outcomes in our study. We also acknowledge that we only measured serum B12. It is possible that our findings based on serum B12 indicates the presence of both bioavailable and non-bioavailable cobalamins. We did not use markers such as holo-transcobalamin, methylmalonic acid, or total plasma homocysteine to improve our definition of B12 status. Having these additional B12 biomarkers used individually or in combination [29] would have been the ideal. One other limitation was the small sample sizes when stratifying into the three B12 status groups, potentially affecting the capacity to detect statistically significant differences.

Further intervention studies are required to establish whether there is a causal relationship between *H. pylori* infection and B12 absorption in locations such as rural Mexico. However, the chronic *H. pylori* infection in these Mexican women is of concern because it may increase their risk of developing peptic ulcers, gastric cancer, and gastric atrophy in the future. Most studies of B12 status in adults have focused on the elderly, since this group has a high prevalence of deficiency even in industrialized countries. We selected younger women to determine whether B12 deficiency was common even before they became elderly. Interestingly age was a positive rather than an inverse predictor of serum B12 concentrations in this younger group, possibly because pregnancy and lactation had caused greater depletion of B12 in the younger women.

The dietary intake estimation for most of the women was at the level of adequacy which we would expect to guarantee adequate status in most of the women. Other possible contributors to the low serum B12 concentrations include bacterial overgrowth [30], lack of intrinsic factor, and possibly polymorphisms in transcobalamin [31,32]. Presumably any of these conditions could exacerbate deficiency due to low intake of the vitamin. However, serum B12 concentrations were directly correlated with the estimated dietary intake of the vitamin and with ASF intake. Although two servings per day were consumed on average, the total estimated weight of ASF consumed (including eggs and dairy products) averaged only 250 g/day, and 34% consumed less than the EAR for B12. Low serum B12 concentrations were more prevalent in those who consumed low amounts of ASFs. In addition to B12 deficiency, approximately 30% of these women had iron deficiency. Serum B12 and ferritin concentrations were significantly although weakly correlated. Co-existing iron and B12 deficiency have been noted in other studies [33,34,35], possibly because ASFs are the best source of both nutrients.

This study is an example of the existing combination of high prevalence of overweight or obesity and micronutrient deficiencies such as B12 and iron deficiency in rural Mexico. The prevalence of obesity was twice that reported by Arroyo et al. in urban Mexican women [36]. Folate status was normal, supporting similar results reported previously in children and women from rural Mexico and Guatemala City [1,2]. It is possible that serum folate adequacy was explained by the dietary folate intake from beans and other foods. In this study, there were no high levels of serum folate, possibly because the majority of the women consumed unfortified home-produced maize (mainly “tortillas”) and were not receiving folic acid from mandatory fortification.

## 5. Conclusions

We conclude that the main or at least a major cause of the low serum B12 concentrations in these Mexican rural women was a low intake of the vitamin due to a low intake of ASFs. *H. pylori* infection was very common and while it did not appear to be a contributor to the low serum B12 concentrations, it should be considered a public health concern. Iron deficiency and gastric inflammation were also major public health issues, but folate deficiency was not. Low serum B12 concentrations were associated with low B12 dietary intake and not with *H. pylori* infection or abnormal gastric function in these rural Mexican women.

## Figures and Tables

**Table 1:** General characteristics of women by serum B12 category.

Variable	Serum B12 Category		
	Low (≤148 pmol/L) *n* = 53	Marginal (148−221 pmol/L) *n* = 59	Adequate (>221 pmol/L) *n* = 79
Age, years	33.4 ± 11.8	34.5 ± 10.9 ^b^	39.5 ± 13.3 ^c^
Weight, kg	66.2 ± 13.3	66.6 ± 15.7	65.6 ± 15.5
Height, cm	151.4 ± 6.0	151.2 ± 5.4	151.4 ± 6.0
Body mass index, kg/m^2^	29.0 ± 6.1	29.1 ± 6.2	28.6 ± 6.3
Diastolic pressure, mmHg	75.0 ± 10.4	74.0 ± 10.4	75.2 ± 10.3
Systolic pressure, mmHg	118.8 ± 15.4	118.8 ± 13.	127.1 ± 43.2
Number of children	4.8 ± 3.4 ^a^	3.8 ± 2.4 ^b^	5.0 ± 3.4
Number of households	5.9 ± 2.4	5.7 ± 2.5	5.8 ± 2.9
Household crowding	3.8 ± 1.7	3.4 ± 1.5	3.5 ± 1.8
Number of bedrooms	1.8 ± 0.8	1.8 ± 0.8	1.9 ± 0.9
Education, years	5.0 ± 3.2	5.5 ± 3.6 ^b^	3.8 ± 3.6 ^c^
Reported income, MXN/months^1^	444.7 ± 262.9	428.3 ± 209.5	408.9 ± 161.4

Data are mean ± SD.^1^ 1 USD = 10.6 Mexican pesos at the time of the survey. Superscripts: “a” indicates that low vs. marginal, “b” indicates that marginal vs. adequate, and “c” indicates that low vs. adequate are significantly different (*p* < 0.05) with one-way ANOVA and Bonferroni’s method as the post hoc test.

**Table 2:** Biochemical and hematological parameters by serum B12 category.

Variable	Serum B12 Category		
	Low (≤ 148 pmol/L) *n* = 53	Marginal (148−221 pmol/L) *n* = 59	Adequate (>221 pmol/L) *n* = 79
B12, pmol/L	85.1 ± 36.9	174.9 ± 21.4	322.1 ± 89.0
Folate, nmol/L	16.6 ± 5.3	16.9 ± 5.2	18.4 ± 8.1
Ferritin, µg/L	39.5 ± 58.4	60.7 ± 85.2	57.9 ± 78.6
Iron deficiency, %	40	30	24
Hb, g/L	132.5 ± 19.3 ^a^	135.5 ± 19.3	138.0 ± 13.2 ^c^
Anemia, %	21	13	21
Hct, %	42.3 ± 5.5	43.0 ± 5.3	43.3 ± 3.9
MCV, fL	86.1 ± 8.8	87.1 ± 9.4	87.8 ± 5.7
MCH, pg	27.0 ± 3.4	27.5 ± 3.7	28.0 ± 2.5
MCHC, g/L	31.3 ± 1.6	31.5 ± 1.7	31.8 ± 1.8
RBC, × 10^6^ cells/µL	4.9 ± 0.4	4.9 ± 0.3	5.0 ± 0.4
WBC, × 10^3^ cells/µL	6.9 ± 1.7	7.1 ± 1.7 ^b^	6.5 ± 1.5
Platelets × 10^3^ cells/µL	237.4 ± 67.7	246.6 ± 69.1	242.6 ± 66.9
Lymphocytes, × 10^3^ cells/µL	34.7 ± 7.6	32.8 ± 6.6	34.0 ± 7.2

Data are mean ± SD or %. Superscripts: “a” indicates that low vs. marginal, “b” indicates that marginal vs. adequate, and “c” that indicates low vs. adequate are significantly different (*p* < 0.05) for one-way ANOVA with Bonferroni’s method as the post hoc test. Abbreviations: Hb, hemoglobin; Hct, hematocrit; MCV, mean corpuscular volume; MCH, mean corpuscular hemoglobin; MCHC, mean corpuscular hemoglobin concentration; RBC, red blood cell count; WBC, white blood cell count.

**Table 3:** Serum gastric function parameters by serum B12 category.

Variable	Serum B12 Category		
	Low (≤ 148 pmol/L) *n* = 53	Marginal (148−221 pmol/L) *n* = 59	Adequate (>221 pmol/L) *n* = 79
Gastrin, pmol/L	57.4 ± 42.0 ^a^	83.8 ± 72.0	76.4 ± 52.8 ^c^
Pepsinogen I, µg/L	50.5 ± 24.1 ^a^	69.5 ± 40.7 ^b^	56.9 ± 30.1 ^c^
*Helicobacter pylori* -positive, %	69	75	68
Gastric function class, %			
Normal	19	27	23
Non-atrophic *H. pylori* infection	46	49	46
Atrophic gastritis of the antrum	0	0	1
Atrophic gastritis of the corpus	2	0	0
Atrophic pangastritis	33	24	31

Data are mean ± SD or %. Superscripts: “a” indicates that low vs. marginal, “b” indicates that marginal vs. adequate, and “c” indicates that low vs. adequate are significantly different (*p* < 0.05) for one-way ANOVA with Bonferroni’s method as the post hoc test. The Chi-squared test was used to compare percentages.

**Table 4:** Dietary intake by serum B12 category.

Variable	Serum B12 Category		
	Low (≤ 148 pmol/L) *n* = 53	Marginal (148−221 pmol/L) *n* = 59	Adequate (> 221 pmol/L) *n* = 79
Total energy, kcal/day	2855.7 ± 901.3	2832.8 ± 841.2	2868.4 ± 690.4
Protein, g/day	84.1 ± 27.9	88.9 ± 29.0	87.3 ± 25.5
Fat, g/day	77.1 ± 26.5	78.4 ± 28.2	84.7 ± 25.2
Carbohydrate, g/day	436.7 ± 134.9	439.5 ± 147.7	432.7 ± 113.1
B12, µg/day	2.7 ± 2.1 ^a^	4.4 ± 4.3	4.1 ± 3.6 ^c^
ASF, servings/day	2.2 ± 1.1	2.3 ± 1.1 ^b^	2.6 ± 1.0 ^c^
Total ASF, g/day	204.5 ± 133.0 ^a^	243.6 ± 141.6 ^b^	299.6 ± 155.2 ^c^
ASF, kcal/day	373.2 ± 208.9	434.8 ± 249.4	474.1 ± 317.8
B12 ASF, µg/day	2.5 ± 2.0 ^a^	4.2 ± 4.2	3.9 ± 3.5 ^c^

Superscripts: “a” indicates that low vs. marginal, “b” indicates that marginal vs. adequate, and “c” indicates that low vs. adequate are significantly different (*p* < 0.05) for one-way ANOVA with Bonferroni’s method as the post hoc test. Abbreviations: ASF, animal source foods.

**Table 5:** Associations between B12 dietary intake and B12 deficiency.

Serum B12 Category		B12 EAR		Total B12 Intake			B12 Intake from ASFs		
		<EAR	≥EAR	T_1_	T_2_	T_3_	T_1_	T_2_	T_3_
Low	OR	2.7 (1.2 − 5.7)	1	3.6 (1.4 − 9.5)	1.9 (0.7 − 4.8)	1	3.0 (1.1 − 7.8)	2.3 (0.9 − 5.9)	1
	*p*	0.01		0.01	0.20		0.02	0.08	
Marginal	OR	1.7 (0.8 − 3.7)	1	1.2 (0.5 − 2.7)	0.5 (0.2 − 1.1)	1	1.1 (0.5 − 2.5)	0.6 (0.6 − 1.4)	1
	*p*	0.16		0.72	0.09		0.81	0.22	

Logistic regressions were run defining the low and marginal serum B12 categories as dependent variables versus B12 dietary intake above and below the EAR, between tertiles of total B12 intake and between tertiles of B12 intake from ASF as independent variables. Abbreviations: EAR, estimated average requirement; OR, odds ratio; T, tertile. Bold values denote statistical significance at *p* < 0.05.
